# The Role of Circular RNAs in Immune-Related Diseases

**DOI:** 10.3389/fimmu.2020.00545

**Published:** 2020-04-02

**Authors:** Rou Xie, Yongxin Zhang, Jun Zhang, Jing Li, Xikun Zhou

**Affiliations:** ^1^State Key Laboratory of Biotherapy and Cancer Center, West China Hospital, Sichuan University and Collaborative Innovation Center for Biotherapy, Chengdu, China; ^2^State Key Laboratory of Oral Diseases, National Clinical Research Center for Oral Diseases, West China College of Stomatology, Chinese Academy of Medical Sciences Research Unit of Oral Carcinogenesis and Management, Sichuan University, Chengdu, China

**Keywords:** circRNAs, immune regulation, immune-related diseases, biomarker, exosomal circRNA

## Abstract

Circular RNAs (circRNAs) are a novel class of RNAs with a covalently closed loop structure without a 3′ polyadenylation [poly-(A)] tail or a 5′ cap. They used to be considered as the occasional and useless products of RNA splicing errors because they could not be detected by traditional RNA sequencing technology. Benefiting from the development of specific biochemical and computational approaches, researchers showed that circRNAs are universally expressed and functional. Further studies have revealed their important functions regarding regulating gene expression at the transcriptional and post-transcriptional levels. These functions include acting as microRNA (miRNA) sponges, binding to RNA-binding proteins (RBPs), acting as transcriptional regulatory factors, and serving as translation templates. The advances in circRNA research has opened researchers' eyes to a new area of research on the roles of circRNAs in the pathogenesis of various diseases, especially at the immune level because of the close relationship between circRNAs and the immune response. Emerging research indicates that circRNAs could act as potential biomarkers related to diagnosis, therapeutic effects, and prognosis, and they may be effective therapeutic targets in immunological disorders, including certain diseases that are currently difficult to treat.

## Introduction

Circular RNAs (circRNAs) are a type of long non-coding RNA (lncRNA) circularized without a 3′ polyadenylation [poly-(A)] tail or a 5′ cap, resulting in a covalently closed loop structure, and they are an important new research topic. circRNAs were first discovered in RNA viruses in the 1970s (1), but only a small number of circRNAs were initially discovered due to limitations in traditional polyadenylated transcriptome analyses. Owing to the development of specific biochemical and computational methods, recent studies have shown that abundant circRNAs occur in eukaryotic transcriptomes naturally and widely. And it has been found that circRNAs play vital roles in many kinds of physiological and pathological processes, such as acting as miRNA sponges, binding to RNA-binding proteins (RBPs), acting as transcriptional regulatory factors, and even serving as translation templates. As a result, circRNAs have become a new research topic, following on from miRNAs and lncRNAs. Researchers consider circRNAs as potential excellent biomarkers related to the diagnosis, therapeutic effects, and prognosis of various diseases, and they may be effective therapeutic targets due to their stability and tissue/development-stage specificity. In this review, we focus on the properties of circRNAs and their immune relationship to disease, revealing the functions of circRNAs in immune-related diseases. Additionally, we investigate several other diseases involving circRNAs to provide a basis for several other directions for future research.

## Overview of circRNAs

### Discovery and Formation of circRNAs

The concept of circRNAs was first reported in 1976 after the discovery of circRNAs in RNA viruses (1). Thereafter, researchers identified the formation of circRNAs in human cells (2–4). But they were considered as the products of RNA splicing errors that lacked biological functions. It is because of their structure, a ring covalently bound by a 5′ cap and 3′ poly (A) tails, which means that there is no 5′ cap or 3′ poly (A) tails. This intrinsic characteristic has enabled them to escape from detection using traditional polyadenylated transcriptome analyses, so there were only a few discoveries of circRNAs in the past.

As time went on, specific biochemical and computational methods were rapidly developed, and many circRNAs were found in various cell lines of different species including but not limited to fungi, plants, insects, and mammals (5–7). Research have revealed that circRNAs are highly represented and naturally occurring in eukaryotic transcriptomes (8, 9). Most circRNAs are derived from known genes and can be divided into three groups according to their constituent sequences: exonic circRNAs, circular intronic RNAs (ciRNAs) and circRNAs composed of exons and introns. Most of them are formed by reverse complementation of introns and exon skipping. Recent studies have shown that back-splicing, the way circRNAs generated by, a type of alternative splicing, requires spliceosomal machinery and can be modulated by both cis-regulatory elements and trans-acting factors (6, 10, 11). Although most of circRNAs have low expression, some circRNAs have been proven to have higher abundance than their linear counterparts in biological samples (9). This may mean that circRNAs have irreplaceable biological functions under physiological conditions. We could reasonably assume that circRNAs play vital roles in various physiological and pathological processes.

### Characteristics of circRNAs

circRNAs are usually more stable than their linear counterparts. Lacking 5′ cap and 3′ poly-(A) tail, circRNAs can resist hydrolysis by various cellular endonucleases (5). It was reported that four circRNAs have half-lives that exceeded 48 h, whereas their linear counterparts' half-lives were shorter than 20 h (5).

Another feature of circRNAs is their high abundance. Although circRNAs used to be discovered sporadically by misdirected analysis techniques, information on more and more circRNAs has been published due to the great progress made related to specific biochemical and computational methods. It has been reported that there are 1,950 circRNAs in human leukocytes, and 1,903 circRNAs in mice (12). Based on RNA-seq, Salzman et al. (13) found a large amount of circular transcripts in both normal cells and cancer cells, with 10% of human genes being able to be transcribed to form circular RNA. The circRNA Sry is even more abundant than its linear counterpart in adult human testes (14). This is probably because circRNAs are stable enough to accumulate, although the backsplicing involved in the biogenesis of circRNAs is commonly less efficient than classical splicing.

The localization of circRNAs also exhibits important characteristics. They are generally located in the cytoplasm and have been identified in exosomes in culture media (15, 16). More notably, the distribution of circRNAs demonstrates tissue and developmental stage specificity (17). For instance, antisense to the cerebellar degeneration-related protein 1 transcript (CDR1as) is highly abundant in the mammalian brain but is expressed at low levels or is even absent in other tissues and organs (18). Although Sry circRNA comprised the largest proportion of X in embryonic brains on days 11–19 after birth, the linear Sry transcripts became increasingly plentiful while the Sry circRNA faded away (19). A total of 10,032 new circRNAs were identified in preimplantation human embryos by circRNA sequencing, most of which were developmentally specific and dynamically regulated (20). This indicates that circRNAs in different locations may have different biological functions.

In addition, circRNAs are evolutionarily conserved (12). For example, the sodium/calcium exchanger protein 1 (NCX1) circular transcript has been identified in humans, mice, rats, rabbits, and monkeys (21). Moreover, the discoveries of circRNAs in fungi, plants, and protists suggest that circRNAs could date back far into the past (22).

### Biological Functions of circRNAs

Although the biological functions of most circRNAs are unclear, the four main functions of circRNAs are discussed below.

**Figure 1:**
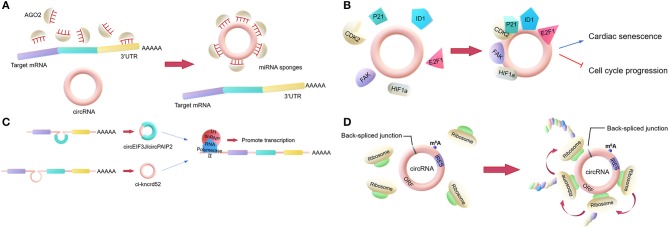
Main biological functions of circRNAs. **(A)** miRNA sponges. circRNAs regulate the activities of mature miRNAs by binding to them, thereby protecting the target mRNA from the miRNA's attack. **(B)** RNA-binding protein (RBP) sponges. Some circRNAs can bind to RBPs. For example, circFoxo3 can bind to certain proteins to regulate cardiac senescence and cell cycle progression. **(C)** Regulator of parent gene transcription. EIciRNAs, such as circEIF3J and circPAIP2, can bind to U1 snRNP to form an EIciRNA-U1 snRNP complex, which interacts with RNA polymerase II to regulate the transcription of parental genes. **(D)** Translation. Some circRNAs containing an IRES can encode proteins via an open reading frame across the back-spliced junction.

The most classic role is to act as a miRNA sponge. This involves some circRNAs regulating the activities of mature miRNAs by binding to the miRNAs and then reducing their ability to target mRNAs. In general, a circRNA that acts as a miRNA sponge has dozens of miRNA binding sites, so it can resemble a sponge, adsorbing many miRNAs (Figure 1A). For example, CDR1as, a circRNA that is highly expressed in the human and mouse brain, has over 60 binding sites for miR-7, which is involved in the regulation of several genes in the brain. Additionally, the Sry circRNA acts as a miRNA sponge for miR-138, with at least 16 binding sites for miR-138 (18, 23).

However, only a very small minority of circRNAs have more than 10 miRNA binding sites, indicating that the miRNA sponge function is not common. Researchers proposed a hypothesis that circRNAs may act as “sponges” for other factors, for example, RBPs (24). Compared to their linear counterparts, bioinformatics analyses of circRNA sequences only found a few binding sites for RBPs in the past. However, nowadays, the interactions between circRNAs and proteins are mainly analyzed by new techniques such as RNA pull-down and RNA immunoprecipitation (RIP). These techniques have shown that certain circRNAs function by binding to proteins. Holdt et al. (25) discovered that a circRNA could bind to the C-terminal lysine-rich domain of pescadillo ribosomal biogenesis factor 1 (PES1), preventing the interaction between PES1 and relevant rRNA and, in contrast to the linear transcript, reducing ribosome biogenesis and inducing apoptosis (25, 26). This is not a unique example, as the circRNA SMARCA5, along with several other circRNAs with QKI-binding sites encoded in the introns, can bind to QKI to regulate circRNA formation during the epithelial–mesenchymal transition (EMT) (27). Another study revealed that circFoxo3 can interact with p21 and CDK2 to inhibit cell cycle progression (28). CircFoxo3 also can bind and inhibit senescence-associated proteins (ID1 and E2F1) and stress-related proteins (HIF1a and FAK) in the cytoplasm to regulate cardiac senescence (Figure 1B) (29). Furthermore, circ-HuR can bind CCHC-type zinc finger nucleic acid binding protein (CNBP), inhibit CNBP-promoted human antigen R (HuR) expression and the development of gastric cancer, and may become a potential therapeutic target for gastric cancer (30).

In addition to the two above functions, circRNAs can also regulate gene transcription directly or indirectly. Firstly, there is a competition between the biosynthesis of circRNAs and linear RNAs, with the expression of linear RNAs being regulated by circRNAs (31, 32). Some circRNAs may act as transcriptional regulators. Exon-intron circRNAs (EIciRNAs) in the nucleus can interact with U1 small nuclear ribonucleoproteins (U1 snRPN), thus promoting the transcription of the parental gene (33). Similarly, ci-ankrd52 interacts with the RNA polymerase II complex to affect its enzymatic activity and upregulate the transcription of the parental gene (Figure 1C) (34). Additionally, the intron-containing circRNAs produced by CUT-like homeobox 1 (CUX1), can bind to EWS RNA-binding protein 1 (EWSR1) to promote MYC-associated zinc finger protein (MAZ) transactivation and leads to transcriptional changes in CUX1 as well as other genes associated with tumor progression (35).

Moreover, according to recent studies, circRNAs can be translated into proteins. Ordinarily, circRNAs cannot be translated. However, as many circRNAs are located in the cytoplasm and contain exon sequences, circRNAs are hypothesized to serve as translation templates in certain conditions (36). It has been proved that circRNAs containing an internal ribosome entry site (IRES) can be translated into proteins (Figure 1D) (37). It was shown that the circRNA AKT3 is translated to a new functional protein, AKT3-174aa, using overlapping codons. *In vivo* and *in vitro* experiments showed that the AKT3-174aa protein negatively regulates the PI3K/AKT signaling pathway by interacting with a key regulatory kinase of the classical PI3K/AKT signaling pathway, PDK1, and inhibits the occurrence and development of brain tumors (38). Furthermore, cardiac tissue translational analysis also strongly indicated that circRNAs can be translated (39).

## circRNAs in Immune Regulation

The immune system is a complex network that maintains the physiological balance and stability of the internal environment of the organism. The hyperactivity of immune regulation will cause damage to the body's own healthy tissues, while hypoactive immune regulation fails to make an effective response against pathogen invasion. Therefore, it is very important to study the immune response and molecular pathway in the immune system. As a newly functional biological macromolecule, how circRNAs play a role in the immune system is of great research significance.

There have been some evidences that circRNAs are directly involved in immune regulation. As early as 2017, Howard Y. Chang reported that cells can recognize intracellular circRNA (endogenous circRNA) and *in vitro* synthesized circRNA (exogenous circRNA) by retinoic acid-inducible gene I (RIG-I), which can activate the autoimmune pathway (40). Then, they further proved that circFOREIGEN or PEI packaging circFOREIGEN both can play a similar role to poly(I: C) *in vivo*, and found that cells can distinguish between endogenous circRNAs and exogenous circRNAs by identifying whether carried N6-Methyladenosine modification (m6A modifications) (41). They further concluded that exogenous circRNAs without m6A modification bind to K63-linked ubiquitin chains (K63-Ubn) and RIG-I and promote RIG-I polymerization and activation, thereby promoting the polymerization of downstream mitochondrial antiviral signal (MAVS) and inducing the dimerization activation of Interferon Regulating Factor 3 (IRF3), and ultimately inducing the expression of genes in the autoimmune pathway.

Another study found that circRNAs can competitively bind double-stranded RNA-activated protein kinase (PKR) to regulate cellular immune signaling pathways extensively (42). Many circRNAs tend to form small imperfect RNA duplexes ranging from 16 to 26bp, which similar to dsRNA structures, bind and inhibit PKR. When cells are processed by poly(I:C) stimulation or viral infection, RNase L activates and thus degrades the circRNAs, then release PKR and activates the downstream antiviral immunity mechanism.

Macrophages are a kind of important immune cells and is an important part of innate immunity. When stimulated by local microenvironment, macrophages will be activated and polarized into different subtypes to play different functions. Macrophages subtypes (M1-type macrophages and M2-type macrophages) play a key role in the induction and regulation of specific immune responses and the development and recovery of disease. It have been indicated that circZC3H4 take part in SiO_2_ induced macrophage activation through HECTD1/ZC3H12A dependent ubiquitin-proteasome pathway (43). Circ-RasGEF1B is necessary for macrophage activation after exposure to lipopolysaccharide (LPS) (44). Zhang et al. (45) found that there were differential expression of 189 circRNAs between M1 and M2 macrophages through microarray analysis techniques. All of which strongly suggested the relationship between circRNAs and macrophage polarization. In addition, the activation of macrophages may produce circRNAs that affect the immune function of macrophages. For example, the circRNA circ-RasGEF1B induced by lipopolysaccharide (LPS) can positively regulate the expression of intercellular adhesion molecule 1 (ICAM-1), an adhesion protein located on the surface of macrophages' cell membrane, in LPS/toll-like receptor 4 (TLR4) signaling pathway, and promote the intercellular interaction during antigen presentation by regulating the stability of mature ICAM-1 mRNA (44). Furthermore, circ-ANRIL may be involved in the apoptosis of macrophages and inhibit the proliferation of macrophages (25). Thus, it can be seen, circRNAs play an important role in the development, activation and function of macrophages.

## circRNAs in Immunological Diseases

circRNAs are involved in a variety of pathophysiological processes based on the above-mentioned functions and may also serve as diagnostic markers or therapeutic targets, especially in immune-related diseases. Treatment of immune diseases mostly involves suppressing the immune response against the body by using immunosuppressants, such as adrenocorticosteroids, or cytotoxic drugs. However, they have a common adverse effect, affecting the body's anti-infection and anti-tumor immunity to varying degrees. Therefore, it is of great clinical significance to develop biological agents and natural inhibitors with little or no adverse effects on immune function. At the same time, there are many diseases directly or indirectly related to the immune system in the pathogenesis, also lacking specific, effective treatments without serious side effects. The discovery of circRNAs has provided new opportunities for the study of these immune-related diseases.

### circRNAs in Immune-Related Diseases

In general, autoimmune diseases develop upon failure of numerous regulatory pathways. The damage to the tolerance of autoantigens is often thought to be the result of genetic and environmental risk factors. The exact mechanisms whereby autoimmune diseases develop are still not well-understood (46) (Table 1). The discovery of circRNAs has opened up a new research avenue in this regard.

**Table 1:** The circRNAs related to immunological diseases.

**Diseases**		**circRNAs**	**Roles in diseases**	**References**
Autoimmune diseases	Rheumatoid arthritis	circRNA-CER	May be upregulated by IL-1 and TNF-α, acts on miR-136 to modulate the expression of MMP13, thus participating in cartilage extracellular matrix injury.	(47)
	Systemic lupus erythematosus	circIBTK	Acts as a miR-29b sponge to inhibit DNA demethylation and AKT signaling in SLE.	(48)
		circRNAs with duplex structures	Bind to and inhibit double-stranded RNA-activated protein kinase R (PKR).	(42)
	Hematologic malignancy	cia-aGAS	Binds to cGAS and inactivates its enzymatic activity to protect dormant hematopoietic stem cells (HSCs) from cGAS-mediated exhaustion, regulating the balance between self-renewal, and differentiation of HSCs.	(49)
	Diabetic nephropathy	circRNA-15698	Acts as a miR-185 sponge and positively regulates the expression of TGF-β1 protein, thus promoting extracellular matrix-related protein synthesis during diabetic nephropathy progression.	(50, 51)
Virus infections	Virus infection	Foreign circRNAs	Sensing of diverse foreign circRNAs may activate the RIG-I-mediated immune response.	(40)
	Antiviral immunity	Endogenous circRNAs	NF90/NF110 binds to circRNAs to form circRNA-protein complexes (circRNPs). circRNA expression decreases upon viral infection, and NF90/NF110 released from circRNP bind to viral mRNAs to play an antiviral role.	(52)
Cancers	Glioma	cZNF292	Inhibiting cZNF292 using small interfering RNA causes cell cycle arrest via the Wnt/β-catenin signaling pathway, thus indirectly restraining glioma cell proliferation.	(53)
	Lung adenocarcinoma	has-circ-0013958	Overexpression of has-circ-0013958 accelerates tumor progression and inhibits apoptosis.	(54)
	Acute myeloid leukemia	has-circ0004277	Expression of has-circ0004277 and its linear transcript (WDR37) were recovered after standard chemotherapy.	(55)
Neurological diesases	Parkinson's disease	CDR1as	Acts as a miR-7 sponge; miR-7 modulates the expression of alpha-synuclein (a protein that always accumulates at the onset of Parkinson's disease).	(56, 57)
	Alzheimer's disease	CDR1as	Reduces the ability of miR-7 to thus upregulate UBE2A (a protein that rapidly decreases in Alzheimer's disease and other neurological diseases).	(58–60)
	Neuropathic pain	circAnks1a	Promotes the nucleation of YBX1 and binding to the VEGFB promoter, thus promoting VEGFB expression, and mediating the physiological process of neuropathic pain.	(61)
Cardiovascular diseases	Myocardial infarction	CDR1as	Acts as a miR-7 sponge; miR-7 can target SP1 and PARP, so CDR1as inhibits the function of miR-7a, and thereby promote apoptosis.	(62–64)
		MFACR	Targets miR-652-3p-MTP18 to mediate cardiomyocyte apoptosis, leads to mitochondrial fission, and eventually promotes the development of myocardial infarction.	(65)
	Heart failure and cardiac hypertrophy	HRCR	Increases the expression of activity-regulated cytoskeleton-associated protein (ARC; an apoptosis repressor with a CARD domain), and inhibits heart failure and cardiac hypertrophy.	(63, 66)
Others	Coronary artery disease	has-circ-0124644	Be a potential novel non-invasive diagnostic or therapeutic biomarker.	(67)
	Myocardial infarction	MICRA	Be a potential novel non-invasive diagnostic or therapeutic biomarker.	(68, 69)
	Type 2 diabetes mellitus	has-circ0054633	Be a potential novel non-invasive diagnostic or therapeutic biomarker.	(70)
	Acute myeloid leukemia	circ-VIM	Be a potential novel non-invasive diagnostic or therapeutic biomarker.	(71)
	Major depressive disorder	has-circRNA-103636	Be a potential novel non-invasive diagnostic or therapeutic biomarker.	(47)

*The circRNAs mentioned in this review and their roles in diseases*.

Liu et al. (72) used high-throughput circRNA microarray analysis to assess the differences in circRNA expression between patients with rheumatoid arthritis (RA) and healthy subjects and identified chondrocyte extracellular matrix-related circRNA (circRNA-CER). circRNA-CER is upregulated by interleukin-1β (IL-1β) and tumor necrosis factor alpha (TNF-α), and it acts on miR-136 to modulate the expression of MMP13, thus participating in the process of cartilage extracellular matrix injury. This indicates that circRNA-CER may be a potential therapeutic target in RA (72). Another study found that nine circRNAs are upregulated and three circRNAs are downregulated in peripheral blood mononuclear cells (PBMCs) of RA patients compared to PBMCs of healthy controls, which may be related to the pathogenesis of RA (73).

Systemic lupus erythematosus (SLE) is a chronic and incurable autoimmune disease. As many studies have indicated that the initiation and progression of SLE is associated with miRNA, circRNAs are probably vital factors in SLE due to their functions as miRNA sponges. Wang et al. (48) found that circIBTK expression was downregulated in SLE and they further revealed that circIBTK served as a miR-29b sponge in order to inhibit DNA demethylation and AKT signaling in SLE (Table 1). An important study showed that many circRNAs tend to form duplex structures ranging from 16 to 26 bp that bind to and inhibit PKR. Artificial overexpression of circRNAs can help reduce PKR activity in PBMCs in SLE patients, which is beneficial for the treatment of SLE and other autoimmune diseases (42) (Table 1).

A recent study also confirmed that circRNAs play roles in bone marrow failure and hematologic malignancy. A circRNA named cia-aGAS binds to cGAS and inactivates its enzymatic activity to protect dormant hematopoietic stem cells (HSCs) from cGAS-mediated exhaustion, thus regulating the balance between self-renewal and differentiation of HSCs. Cia-cGAS might be a potent suppressor of cGAS-mediated recognition and autoimmune responses. Cia-cGAS deficiency increases type I interferons in the bone marrow and decreases dormant long-term hematopoietic stem cells (LT-HSCs), which disrupts the balance, leading to severe pathologic consequences such as bone marrow failure and hematologic malignancy (49) (Table 1).

A study on circRNA expression profiles in diabetic nephropathy and the effect of circRNAs in mesangial cells also found that circRNA-15698 acted as a miR-185 sponge and positively regulated TGF-β1 expression, thus promoting extracellular matrix-related protein synthesis in diabetic nephropathy progression. Moreover, TGF-β1 is the key factor underlying fibrosis in multiple tissues and organs, indicating that circRNA-15698 might be associated with fibrosis (50) (Table 1).

### circRNAs in Virus Infections

circRNAs have also been shown to have vital roles in virus infections, providing a new strategy for developing vaccines against RNA viruses. Cells may sense diverse foreign circRNAs, such as the circRNAs in hepatitis D virus (HDV) and viroids, and then activate RIG-I-mediated immune response. The transfection of purified circRNAs generated *in vitro* into mammalian cells can induce the expression of innate immunity genes and thus protect against viral infection. This suggests that cells can distinguish self and foreign circRNAs, which is dependent on the intron encoding the circRNAs (40) (Table 1).

In another study, Chen et al. (40) found that the antiviral effect of NF90/NF110 was related to the expression of endogenous circRNAs. NF90/NF110 binds to circRNAs to form circRNA-protein complexes (circRNPs). The expression of circRNAs decreases during viral infection, so NF90/NF110 is released from circRNPs and binds to viral mRNAs to play an antiviral role. Li et al. (52) suspected that the antiviral function is universal among circRNAs, as both endogenous circRNAs and artificial analogs could bind to NF90/NF110 (Table 1).

### circRNAs in Cancers

Using deep RNA-seq technology, researchers identified many circRNAs related to the clinicopathological features of cancer, such as tumor node metastasis (TNM) stages, and recurrence. Many studies have verified that circRNAs are closely related to cancer initiation and development. Using the UROBORUS tool to analyze RNA-seq data from 27 gliomas and 19 normal brain samples, one study revealed that more than 476 circRNAs were differentially expressed between gliomas and healthy controls (53). cZNF292 was further studied because of its large upregulation in glioma cell lines. Inhibiting cZNF292 using small interfering RNA caused cell cycle arrest via the Wnt/β-catenin signaling pathway, thus indirectly inhibiting glioma cell proliferation (53) (Table 1). Additionally, the miR-134 sponge has-circ-0013958 was significantly upregulated in lung adenocarcinoma patients. The overexpression of has-circ-0013958 accelerated tumor progression and inhibited apoptosis (54). Moreover, lung cancer patients with high expression of circRNA-100876 (encoded by RNF121) had shorter survival times compare to lung cancer patients with lower expression (74). By analyzing 115 human samples of acute myeloid leukemia (AML), Li et al. (55) identified a significantly downregulated circRNA called has-circ0004277 and showed that its expression and the expression of its linear transcript (WDR37) could be recovered after standard chemotherapy, providing new clues for developing clinical chemotherapy (Table 1). In addition, fusion circRNAs (f-circRNAs) generated from chromosomal rearrangements can act as proto-oncogenic factors in tumor cells (10).

### circRNAs in the Neurological Diseases

The nervous system includes the central nervous system and the peripheral nervous system. A large number of studies have found that the occurrence of various neurological diseases is closely related to neuroinflammation, which is caused by infections, trauma, toxic metabolites, and autoimmune abnormalities (75–77). Previous research suggested that circRNAs are widely distributed in the mammalian brain, at even higher abundance than the abundance of corresponding linear transcripts (7). Thousands of circRNAs are upregulated in the mammalian brain during neuronal differentiation, indicating that circRNAs are potentially involved in neurological diseases (78, 79). As mentioned above, CDR1as is an endogenous circRNA that is highly expressed in both the human and mouse brain, acting as a miR-7 sponge. MiR-7 can modulate the expression of alpha-synuclein, a protein that always accumulates at the onset of Parkinson's disease (56, 57) (Table 1). CDR1as may be a therapeutic target in Parkinson's disease. Additionally, CDR1as contributes to Alzheimer's disease by binding to miR-7, reducing the ability of miR-7 thus upregulate ubiquitin-conjugating enzyme E2 A (UBE2A), a protein that decreases rapidly in Alzheimer's disease and other neurological diseases (58, 59) (Table 1). Moreover, CDR1as inhibits the translation of nuclear factor (NK)-kB, indirectly impairs the expression of ubiquitin carboxyl-terminal hydrolase L1, and ultimately causes the degradation of amyloid precursor protein (APP) and β-site APP-cleaving enzyme 1 (BACE1), proteins that underlie the development of Alzheimer's disease (60) (Table 1).

In depressive disorders, circRNAs also play an important role. It was reported that circAnks1a can promote the nucleation of YBX1 and bind to the vascular endothelial growth factor B (VEGFB) promoter, thus promoting VEGFB expression. VEGFB expression can also be regulated by competitive binding of miR-324-3p. Both mechanisms work together to promote VEGFB expression, which ultimately mediates the physiological process of neuropathic pain (61) (Table 1). Moreover, circRNAs are associated with myotonic dystrophy and amyotrophic lateral sclerosis (ALS) (80).

### circRNAs in the Cardiovascular Diseases

Cardiovascular disease (CVD) is the world's leading cause of death, causing immense health and economic burdens on society (81). Various endogenous and exogenous injury factors can stimulate the innate immune response of cardiovascular system, cause a large number of immune inflammatory response, cause tissue damage of cardiovascular system, accelerate the progress of cardiovascular disease. However, despite the increasing incidence of CVD, treatments remain limited (82). Many experiments have shown that the expression of circRNAs is different in CVD, and some experiments have found that circRNAs play important roles in CVD pathogenesis (83). The miR-7 sponge CDR1as is associated with myocardial infarction, as miR-7a/b protects the cardiac pericardium. MiR-7 targets trans-acting transcription factor 1 (SP1) and poly-ADP-ribose polymerase (PARP), could inhibit the ability of miR-7a in reducing apoptosis. Thus, CDR1as certainly induces myocardial infarction to some degree (62–64) (Table 1). Another circRNA, mitochondrial fission and apoptosis-related circRNA (MFACR), targets miR-652-3p-MTP18 to mediate cardiomyocyte apoptosis, leads to mitochondrial fission, and ultimately promotes the development of myocardial infarction (65) (Table 1). MiR-223 plays important roles in the function of vascular smooth muscle cells (VSMCs) and atherogenesis, and researchers found that the miR-223 sponge known as heart-related circRNA (HRCR) increases the expression of activity-regulated cytoskeleton-associated protein (ARC), an apoptosis repressor with a CARD domain, and thereby inhibits heart failure and cardiac hypertrophy (63, 66) (Table 1).

### circRNAs as Biomarkers in Immunological Diseases

Due to the stability and widespread distribution in many human body fluids, such as saliva and blood, circRNAs are considered to be effective biomarkers for disease detection (15, 84, 85). According to a study by Zhao and colleagues, has-circ-0124644 could be a diagnostic biomarker for coronary artery disease, allowing convenient and cheap early diagnosis (67) (Table 1). Similarly, myocardial infarction-associated circRNA (MICRA) could be used as a peripheral blood biomarker to diagnose left ventricular dysfunction in patients with acute myocardial infarction (68, 69) (Table 1). Another study has showed that a circRNA, has-circ0054633, is a potential diagnosis biomarker in prediabetes and type 2 diabetes mellitus (70) (Table 1). Levels of circ-vimentin (circ-VIM) are positively correlated with the number of white blood cells (WBC), lower overall survival (OS), and leukemia-free survival (LFS) in AML patients, which means circ-VIM can be an ideal biomarker for the progression and prognosis of AML (71) (Table 1). What's more, in the peripheral blood, has-circRNA-103636 can be a potential novel non-invasive biomarker related to diagnosis and therapeutic effects in major depressive disorder (MDD), based on its differential expression between MDD patients and controls and its downregulation after 8-week treatment in MDD patients (47) (Table 1).

Exosomes are nanoscale vesicles of 30–150 nm in size that contain proteins, mRNAs, and miRNAs. They contribute to communication between cells, and they can be secreted by a variety of cells, such as T cells, dendritic cells, tumor cells, mesenchymal stem cells, and endothelial cells, and so on. circRNAs are enriched in exosomes and can be transported by exosomes. It is reported that cancer-associated circRNAs were detected in tumor-derived exosomes in colon cancer and leukemia patients (10, 86). The level of circRNAs in cells would be significantly reduced if exosomes were removed (15). As exosomes transfer circRNAs to distant sites, exosomal circRNAs may play important roles in disease progression. Like in gastric cancer (GC), circRNAs in plasma exosomes have specific expression characteristics. In particular, ciRS-133 is associated with the browning of white adipose tissue (WAT) in GC patients. GC cell exosomes deliver ciRS-133 to preadipocytes, which promotes the differentiation of preadipocytes into brown-like cells by activating PRDM16 and inhibiting miR-133, participates in WAT browning, and plays a key role in cancer-related cachexia (87). Additionally, FLI1 exonic circRNA (FECR) in serum exosomes, a new oncogenic driver, is associated with poor survival and poor clinical response to chemotherapy and promotes tumor metastasis through the mir584-ROCK1 pathway (88).

Besides, as exosomes can be detected in a variety of body fluids, including plasma, urine, saliva, breastmilk, bronchial lavage, cerebrospinal fluid, and amniotic fluid, circRNAs in exosomes also can be more effectively biomarkers for disease diagnosis and prognosis.

## Conclusions and Perspectives

Research has revealed that circRNAs are a class of endogenous RNAs that occur naturally. Mechanistic and functional studies have shown that circRNAs are not simply useless by-products. Our current knowledge of circRNAs is still poor, and they are worthy of further research. As their various functions are being discovered, circRNAs show great research potential, similar to miRNAs and lncRNAs. Studying the relationships between circRNAs and diseases has important clinical implications, including but not limited to discoveries related to pathological roles, diagnostic potential, and therapeutic effects. Due to the close relationships between circRNAs and immune cells, circRNAs must have certain associations with immunological diseases.

The miRNA sponge function of circRNAs indicates a “circRNA-miRNA-mRNA” pathway in immunological diseases. Additionally, circRNAs can function in diseases by binding to proteins and regulating transcription. Moreover, serving as translation templates is an important part of their function. Furthermore, due to the stability and widespread distribution of circRNAs in human body fluids, such as saliva and blood, and especially in exosomes, circRNAs are expected to be effective biomarkers for disease diagnosis. For example, has-circRNA-103636 in peripheral blood is a potential novel non-invasive biomarker related to diagnosis and therapeutic effects in MDD (47), has-circ-0124644 is a potential diagnostic biomarker for coronary artery disease (67), and has-circ0054633 is a potential diagnostic biomarker for prediabetes and type 2 diabetes mellitus (70). Although many previous studies only went as far as exploring the expression changes of circRNAs between patients and healthy individuals, their results do provide a basis for future research.

**Figure 2:**
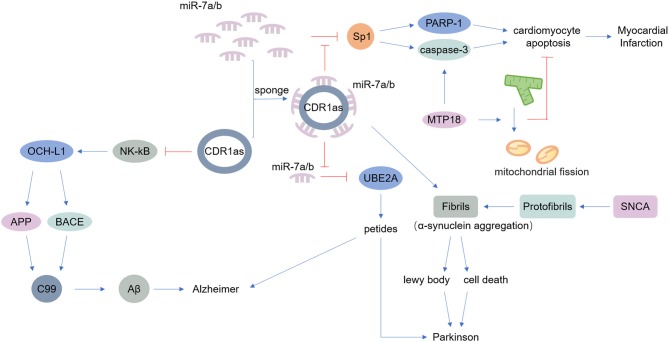
Main known functions of CDR1as. CDR1as can act as a miR-7 sponge. miR-7 modulates the expression of alpha-synuclein, a protein that accumulates at the onset of Parkinson's disease. Additionally, CDR1as reduces the ability of miR-7 to downregulate UBE2A, contributing to Alzheimer's disease and other neurological diseases. Furthermore, miR-7 targets SP1 and PARP; thus CDR1as inhibits the function of miR-7a and thereby promotes apoptosis, inducing myocardial infarction.

However, most studies on the therapeutic effects of circRNAs are currently only at the animal research stage. The development of therapeutics for clinical application still has a very long way to go. It should be noted that a single circRNA may be related to more than one disease, such as CDR1as have been linked to Parkinson's disease, Alzheimer's disease and myocardial infarction (Figure 2). And like CircFoxo3 can interact with p21 and CDK2, also can bind senescence-associated proteins and stress-related proteins. The relationship between them is competition or collaboration? Additionally, although some circRNAs have been characterized, the functions of most circRNAs are still unknown. Similar to mRNAs and lncRNAs, the exploration of the functions of circRNAs must not stop at the current stage. Due to the limitations of current research techniques, research approaches for circRNAs are not mature. Various techniques have been applied to the study of circRNAs, such as RNA-centric techniques and RNA pull-down techniques (89). It is expected that more functions will be discovered and better understanding of various diseases at the genetic level will be developed. Moreover, the characteristics of circRNAs themselves have not been fully revealed, and there are few studies on the mechanisms of circRNA degradation. As the evidence regarding the translation of certain circRNAs continues to accumulate, the types of proteins encoded by circRNAs and the pathways that these proteins are involved in will go far beyond the existing discoveries. The functions of circRNAs are significant, and we have a lot more research to do.

## Author Contributions

All authors wrote the manuscript. RX designed the figures. RX, YZ, and JZ collected the included references and edited the manuscript. JL and XZ provided guidance and revised the manuscript. All authors approved the final manuscript.

### Conflict of Interest

The authors declare that the research was conducted in the absence of any commercial or financial relationships that could be construed as a potential conflict of interest.

## Abbreviations

ALS: amyotrophic lateral sclerosis
APP: amyloid precursor protein
ARC: activity-regulated cytoskeleton-associated protein
AML: acute myeloid leukemia
BACE1: β-site APP-cleaving enzyme 1
CNBP: CCHC-type zinc finger nucleic acid binding protein
CUX1: CUT-like homeobox 1
CVDs: cardiovascular diseases
circRNA-CER: chondrocyte extracellular matrix-related circRNA
circRNAs: circular RNAs
circ-VIM: circ-vimentin
EWSR1: EWS RNA-binding protein 1
EIciRNAs: Exon-intron circRNAs
f-circRNAs: fusion circRNAs
FECR: FLI1 exonic circRNA
HuR: human antigen R
HDV: hepatitis D virus
HSCs: hematopoietic stem cells
K63-Ubn: K63-linked ubiquitin chains
ICAM-1: intercellular adhesion molecule 1
LFS: leukemia-free survival
IL-1: interleukin-1
IRES: internal ribosome entry site
LPS: lipopolysaccharide
IRF3: Interferon Regulating Factor 3
lncRNAs: long non-coding RNAs
LT-HSCs: long-term hematopoietic stem cells
MDD: major depressive disorder
MAZ: MYC-associated zinc finger protein
MAVS: mitochondrial antiviral signal
MFACR: mitochondrial fission and apoptosis-related circRNA
MICRA: myocardial infarction-associated circular RNA
miRNAs: microRNAs
MMP13: matrix metallopeptidase 13
NCX1: sodium/calcium exchanger protein 1
m6A modifications: N6-Methyladenosine modification
OS: overall survival
PARP: poly-ADP-ribose polymerase
PBMCs: peripheral blood mononuclear cells
PES1: pescadillo ribosomal biogenesis factor 1
PKR: double-stranded RNA-activated protein kinase R
poly-(A): polyadenylation
RA: rheumatoid arthritis
RBPs: RNA-binding proteins
RIP: RNA immunoprecipitation
RIG-I: retinoic acid-inducible gene I
RNF121: ring finger protein 121
SLE: systemic lupus erythematosus
snRPN: small nuclear ribonucleoprotein
sp1: trans-acting transcription factor 1
TGF-β1: transforming growth factor-beta 1
TLR4: toll-like receptor 4
TNF-α: tumor necrosis factor alpha
UBE2A: ubiquitin-conjugating enzyme E2 A
VEGFB: vascular endothelial growth factor B
VSMC: vascular smooth muscle cell
WAT: white adipose tissue
WBC: white blood cells
WDR37: WD repeat domain 37.
